# Rationale and Outcomes of Cryoballoon Ablation of the Left Atrial Posterior Wall in Conjunction with Pulmonary Vein Isolation

**DOI:** 10.19102/icrm.2021.120801

**Published:** 2021-08-15

**Authors:** Arash Aryana

**Affiliations:** ^1^Dignity Health Heart and Vascular Institute, Sacramento, CA, USA; ^2^Cardiac Catheterization Laboratory, Mercy General Hospital, Sacramento, CA, USA

**Keywords:** Catheter ablation, cryoablation, cryoballoon, persistent atrial fibrillation, posterior wall isolation

## Abstract

There is strong evidence in support of pulmonary vein isolation (PVI) with concomitant left atrial (LA) posterior wall (PW) isolation (PWI) for the treatment of patients with persistent atrial fibrillation (persAF). While this may be achieved using surgical and catheter-based strategies, there is growing interest in performing this approach using the cryoballoon. There are several potential advantages to this strategy. First, lesions created using the current-generation cryoballoons are typically large and durable. Second, cryoballoon ablation offers a simple technique to directly ablate and debulk the LAPW. Moreover, some consider cryoenergy a safer modality specifically with regard to collateral structures (ie, the esophagus). Based on the available data, cryoballoon PVI + PWI is associated with greater intraprocedural AF terminations and reductions in long-term AF recurrence (typically by ~20%), as compared to PVI alone in patients with persAF, but with similar rates of adverse events. As such, PVI + PWI has emerged as a significant predictor of freedom from recurrent AF (odds ratio: 3.67, 95% confidence interval: 1.44–9.34; p = 0.006) as well as all atrial arrhythmias (hazard ratio: 2.04, 95% confidence interval: 1.15–3.61; p = 0.015). Adjunct radiofrequency ablation to complete PWI is required in at least one-third of the patients, and this need is highly predicted by the LA size (significantly increased with an LA diameter > 48 mm). LAPW reconnection also seems to be associated with LA dimension, particularly an LA diameter greater than 48 mm (negative predictive value: 89.7%). Nevertheless, based on the analysis of patients who underwent repeat electrophysiology study for arrhythmia recurrences, cryoballoon PVI + PWI yields acceptable long-term durability (> 80%).

## Introduction

**Figure 1:**
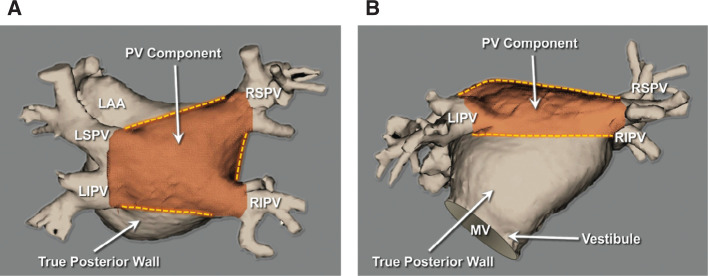
The PV component. **A:** A cardiac computed tomography image showing a posterior projection of the LAPW, including the PVs and the tissue that lies between them—the so-called PV component. **B:** On the other hand, the true LAPW spans from the inferior borders of the inferior PVs to the superior margin of the vestibule, the region surrounding the mitral valve orifice. As such, the PV component in essence represents more the dome of the LA than the PW. LAA: left atrial appendage; LIPV: left inferior pulmonary vein; LSPV: left superior pulmonary vein; MV: mitral valve; PV, pulmonary vein; RIPV: right inferior pulmonary vein; RSPV: right superior pulmonary vein.

Pulmonary vein (PV) isolation (PVI) remains an effective treatment strategy for paroxysmal atrial fibrillation (AF).^1^ However, PVI by itself is often insufficient for the treatment of patients with persistent AF (persAF),^2,3^ with success remaining low despite the various ablation techniques.^4^ Observations from surgical ablation of the left atrial (LA) posterior wall (PW) have implicated the region lying between the PVs in patients with persAF, the so-called PV component^5^ **(Figure 1)**. This is plausible as persAF is generally not considered a triggered dysrhythmia but a substrate-based arrhythmia.^6^ The original Cox maze procedure as well as all its current iterations, which encompass the isolation of this region of the LAPW, have reported improved efficacy even in those with long-standing persAF.^7,8^ Likewise, recent catheter ablation studies^9–15^ have found similar benefits in patients with persAF associated with PW isolation (PWI) within the region of the PV component. Anatomically, this region is defined by the LA roof, superiorly, the left and the right PVs, laterally, and the plane extending from the lower borders of the left and the right inferior PVs inferiorly.^5^ There is clinical, anatomic, and electrophysiologic evidence to suggest that this region of the LAPW may contribute to the genesis and maintenance of AF, particularly in those with persAF. Moreover, targeting this region of the LA inherently represents an extended form of wide-area antral PVI, which too has been shown to be superior to a distal/ostial PVI strategy.^16^

## Anatomic and embryologic evidence supporting catheter ablation of the pulmonary venous component

**Figure 2:**
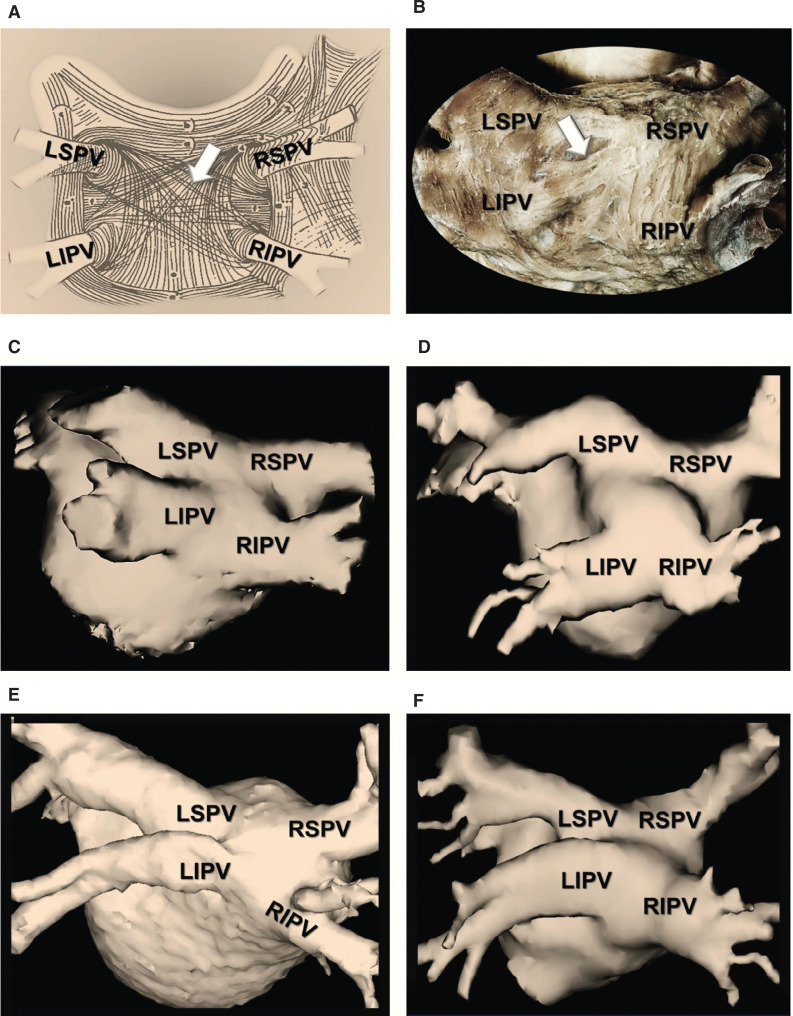
Anatomical examination of the LAPW and the PVs. **A:** An artist’s rendition illustrating the orientation of the myofibrils of the LAPW and the PVs created based on histopathological examinations. As seen, circular muscular fibers arising from the LA (arrow) wrap around the PV ostia forming sphincter-like structures, whereas other fibers extend over the PVs as myocardial sleeves. As such, no definite anatomical boundaries can be identified to clearly demarcate the PV ostia versus antra from the remaining LAPW (adapted from Nathan and Eliakim^17^). **B:** A gross anatomical examination of the myofibrils that cover the LAPW and the PVs in the human, highlighting the same observations (courtesy of A. E. Becker). **C–F:** Cardiac computed tomography images obtained in patients with AF, illustrating variations in LA/PV anatomy. Arguably, in all these cases, a clear and precise boundary between the LAPW and the PVs remains obscure, further alluding that the PV component may in fact be a direct extension of the PVs, themselves. LIPV: left inferior pulmonary vein; LSPV: left superior pulmonary vein; RIPV: right inferior pulmonary vein; RSPV: right superior pulmonary vein.

A visual examination of the PV component and the orientation of its myofibrils suggests direct continuity between this region and the PV antra, as does a gross anatomical assessment of certain LA morphologies **(Figure 2)**, further supporting the notion proposed by some that the PV component may in fact be a direct extension or a constituent of the PV tissue.^5,18^ The PV component forms the LA dome with the superior PVs situated anteriorly and the inferior PVs more posteriorly. Thus, what is commonly referred to by many as the LAPW, in reality, represents the PV component, whereas the true PW extends from the lower borders of the inferior PVs to the superior margin of the vestibule—that is, the region surrounding the mitral valve orifice.^5^ The PV component shares a common primordial origin with the PVs.^5^ The embryologic origin of the four PVs and the PV component can be traced back to the mediastinal myocardium derived from a mid-pharyngeal strand at six weeks of gestation.^19^ As such, the cellular lineage of the PVs and the PV component is entirely different from that of the remainder of the myocardium, which originates from the primary heart tube and the systemic venous tissue.^5,19^

## Electrophysiologic evidence supporting catheter ablation of the pulmonary venous component

As discussed, the PV component is derived from tissues other than the primitive cardiac tube.^19,20^ As such, it is believed to be related more to the PVs than the atrial tissue. Some studies have suggested that the cells from these tissues share more in common with the sinoatrial nodal myocytes, for instance, displaying higher diastolic calcium contents and propensity to spontaneous depolarization.^21^ Moreover, the rationale for ablation of the PV component is further supported by clinical and cellular data, which evoke that this region of the LAPW exhibits significant remodeling believed to play a critical role in the pathogenesis of persAF,^22^ as well as conduction abnormalities,^23^ a higher incidence of delayed afterdepolarizations and larger late sodium and intracellular and sarcoplasmic reticulum Ca^++^ contents, but smaller inward rectifier potassium currents^24,25^ and a reduced resting membrane potential.^25,26^ The LAPW and the PV myocytes exhibit shorter action-potential durations and slower phase 0 upstroke velocities.^26^ Furthermore, the increased duration of AF observed in those with persAF is characterized by reduced wave conduction velocities and complex local activation within the PV component.^27^ The region of the PV component is also believed to be the site of potential collision of activation wavefronts as they sweep across the LA dome.^5^ Anatomically, there seems to be significant heterogeneity in myocardial fiber orientation within the PV antra and the LAPW, creating nonuniform anisotropic conduction favoring local reentry.^28^ Furthermore, the PV component seems to exhibit greater conduction heterogeneity and anisotropy in patients with persAF as compared to those in sinus rhythm. Mapping of fibrillatory waves during cardiac surgery in patients with persAF has revealed simultaneous propagation of longitudinally dissociated fibrillation waves which are separated by continuously changing lines of block.^4^ These lines of block are most densely packed within the PV component, leading to the highest degree of block and dissociation and the lowest incidence of wavefront boundaries formed by collision.^29^ Along these lines, Mandapati et al. found this region of the LA to be responsible for 80% of high-frequency rotors in an isolated sheep heart model.^30^ Favorable conditions for rotor formation include: (1) triggers from the PVs; (2) anisotropy and abrupt changes in wall thickness in the LAPW that create a source–sink mismatch, leading to wave break/reentry; (3) shorter local refractory periods that allow high-frequency driving rotors to persist; and (4) fibrosis that serves as a unidirectional block, which favors rotor formation and acts as an anchor for rotor maintenance.^31^ Similarly, mapping in humans often localizes stable rotors or focal sources^32^ as well as complex fractionated electrograms^33^ in the LAPW and the roof. The PV component has in fact been shown to be a common source of triggers, accounting for approximately 40% of non-PV triggers in patients with AF.^34^

The PV component is also the site of the main autonomic ganglionic plexi related to the LA dome (ie, the superior LA ganglionated plexus), which is believed to modulate extrinsic cardiac innervation and facilitate the occurrence of AF in a hyperactive autonomic state.^5,28^ Thus, it is believed that catheter ablation of the PV component may greatly attenuate the input of these plexi to the PVs and interrupt the vagosympathetic input to the ligament of Marshall and the inferior left ganglionated plexus, which have also been highly implicated in the pathogenesis of AF.^5,28^ In addition to these findings, an elevated LA pressure is believed to disproportionately affect the PV component. Prior studies have implicated acute, rapid LA activation in stretch-related AF with the level of spatiotemporal organization correlating with the degree of pressure elevation.^35^ Moreover, areas of the LA exposed to the highest wall stress, particularly those around the PV antra and the PV component, have been correlated with low voltage and electrical scarring in prior mapping studies.^36^ Accordingly, pathologic studies have similarly found increased fibrosis within the PV component of the LA and the LAPW of patients with chronic AF and mitral valve disease.^23^

## Available techniques for the ablation of the pulmonary venous component

**Figure 3:**
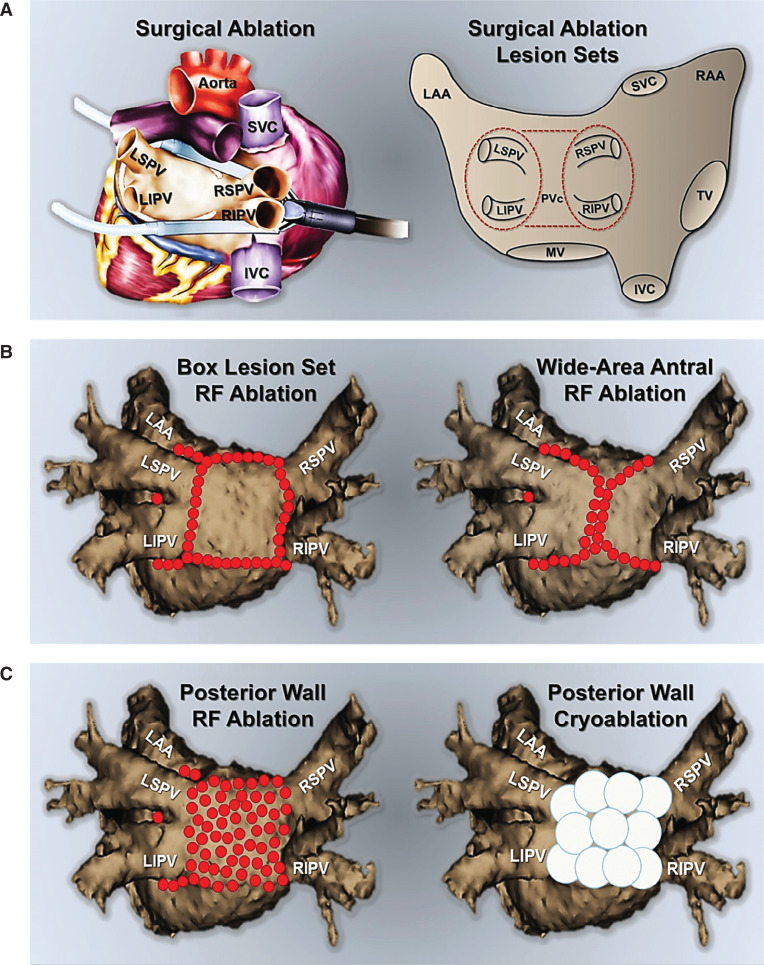
Minimally invasive techniques for the ablation of the LAPW. **A:** One technique involves a surgical approach, using endoscopically delivered epicardial lesions. The catheter-based strategies include, **B:** creating large/wide antral lesions to include the PV component **(left panel)** or a separate LAPW “box” lesion set using point-by-point radiofrequency **(left panel)** or **C:** direct LAPW ablation and debulking using point-by-point radiofrequency **(right panel)**, or the cryoballoon **(right panel)**. IVC: inferior vena cava; LAA: left atrial appendage; LIPV: left inferior pulmonary vein; LSPV: left superior pulmonary vein; MV: mitral valve; PVc: pulmonary vein component; RAA: right atrial appendage; RIPV: right inferior pulmonary vein; RF: radiofrequency; RSPV: right superior pulmonary vein; SVC: superior vena cava; TV: tricuspid valve.

**Figure 4:**
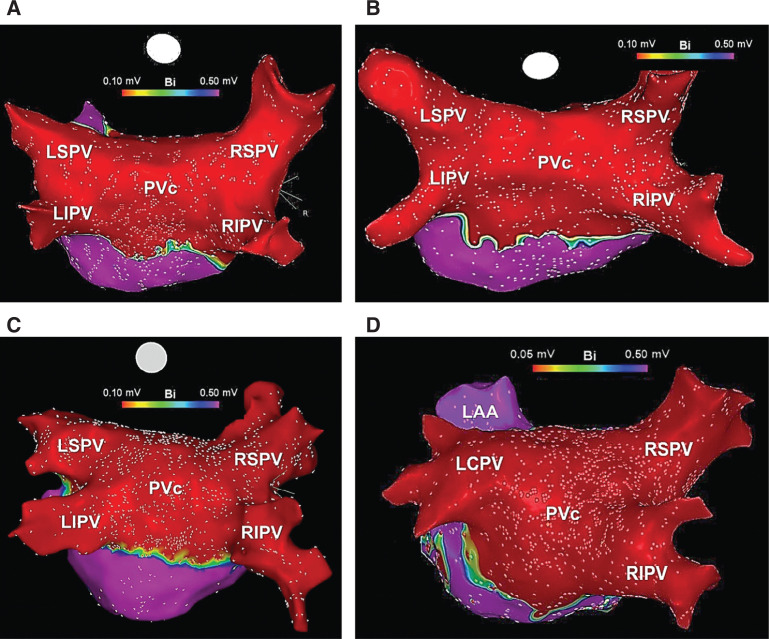
Three-dimensional electroanatomic voltage maps demonstrating PVI + PWI within the region of the PVc performed using the cryoballoon **(A–D)**. This approach has been shown to yield significant LA debulking and offers consistent and incremental benefit over PVI alone, in patients with persAF. LAA: left atrial appendage; LIPV: left inferior pulmonary vein; LSPV: left superior pulmonary vein; PVc: pulmonary vein component; RIPV: right inferior pulmonary vein; RSPV: right superior pulmonary vein.

A variety of minimally invasive strategies have been employed for ablation/isolation of the LAPW and the PV component. One method involves surgical ablation using endoscopically delivered epicardial lesions **(Figure 3A)**. However, this typically warrants an electrophysiology study, either concurrently or during follow-up, to carefully examine the LA voltage and/or to complete PVI + PWI, hence, commonly known as a hybrid approach.^37^ Several catheter-based methods have also been described. One technique involves creating large antral lesion sets to include the PV component versus creating a separate LAPW “box” lesion set using point-by-point radiofrequency ablation **(Figure 3B)**. Though feasible,^38^ the theoretical advantages of this approach are sometimes offset by the challenges of creating uninterrupted, linear LAPW radiofrequency lesions.^39,40^ As such, in many instances, additional applications inside the “box” are required to complete PWI. Another approach involves directly ablating the PW/PV component in conjunction with circumferential PVI using point-by-point radiofrequency or cryoballoon ablation **(Figure 3C)**. The latter can result in significant LA debulking **(Figure 4)**, and seems to offer consistent and incremental clinical benefits in patients with persAF.^11,12^ Meanwhile, prior studies have shown that isolating the PV component not only does not compromise the contractile function of the LA^41–43^ but, in fact, this approach is associated with LA size reduction and reverse remodeling.^44^

## Rationale for cryoballoon ablation of the left atrial posterior wall and the pulmonary venous component

Ablation lesions created using the currently available cryoballoons (second-generation or later) are typically large^45–47^ and durable.^48^ As such, these characteristics make these catheters potentially an attractive tool for performing LAPW ablation. Moreover, some have considered the cryoballoon a safer means for this approach, particularly with regard to certain collateral structures such as the esophagus.^49–51^ Despite the weak level of evidence, there are limited data available from a handful of studies which have examined the effects of cryoballoon versus other ablation modalities on esophageal injury.^50,51^ Based on indirect comparisons, the incidence of esophageal injury/ulceration following AF ablation as confirmed by endoscopy may be as high as 48% to 60% with radiofrequency,^52–54^ but typically only half as prevalent (0%–22%) with cryoballoon ablation.^55–58^ A comparative study^50^ directly evaluating the safety of radiofrequency versus cryoablation on the calf esophagus found that esophageal lesion width and volume were significantly larger with radiofrequency versus cryoablation at 7 days. These authors also detected significant histological differences between the two modalities, including a higher incidence of partial- and full-thickness esophageal ulcerations with radiofrequency versus cryoablation. Furthermore, an interesting study by Cai et al.^59^ recently discovered a correlation between esophageal contraction and cryoenergy, suggesting perhaps that this phenomenon might in itself serve as a protective mechanism to the esophagus during cryoablation. But before recognizing the cryoballoon as a suitable tool for LAPW ablation, it is important to first review the rationale for this approach as well as the biophysics of cryoballoon ablation.

### The level and extent of posterior vein isolation using cryoballoon ablation

The level of PVI achieved using cryoballoon ablation has been the subject of controversy. Reddy et al.^60^ were the first to investigate the level of PVI achieved during cryoballoon ablation of AF. In their study, they examined the precise location of the lesions created using a 23-mm first-generation cryoballoon (Arctic Front; Medtronic, Minneapolis, MN, USA) in a cohort of patients using three-dimensional (3D) electroanatomic mapping. These authors found that isolation had occurred predominantly at the level of the PV ostia, whereas the PV antra were left largely intact/unablated. In a subsequent study, Chierchia et al.^61^ also evaluated the extent and level of PVI in a similar fashion using both 23- and the 28-mm first-generation cryoballoons. They noted that PVI occurred more proximally using the latter, resulting in significantly larger areas of atrial tissue ablation using the 28-mm (40.2% ± 3.9%) versus the 23-mm (20.7% ± 2.8%) balloon. Most recently, Kenigsberg et al.^62^ reported a more contemporary experience using exclusively a 28-mm second-generation cryoballoon (Arctic Front Advance; Medtronic). These authors found that the level of PVI was much wider and more antral than previously reported. Moreover, they noticed partial isolation and debulking of the LAPW by approximately 70% in a cohort of patients who largely exhibited paroxysmal AF and only mild-to-moderate LA enlargement.

**Figure 5:**
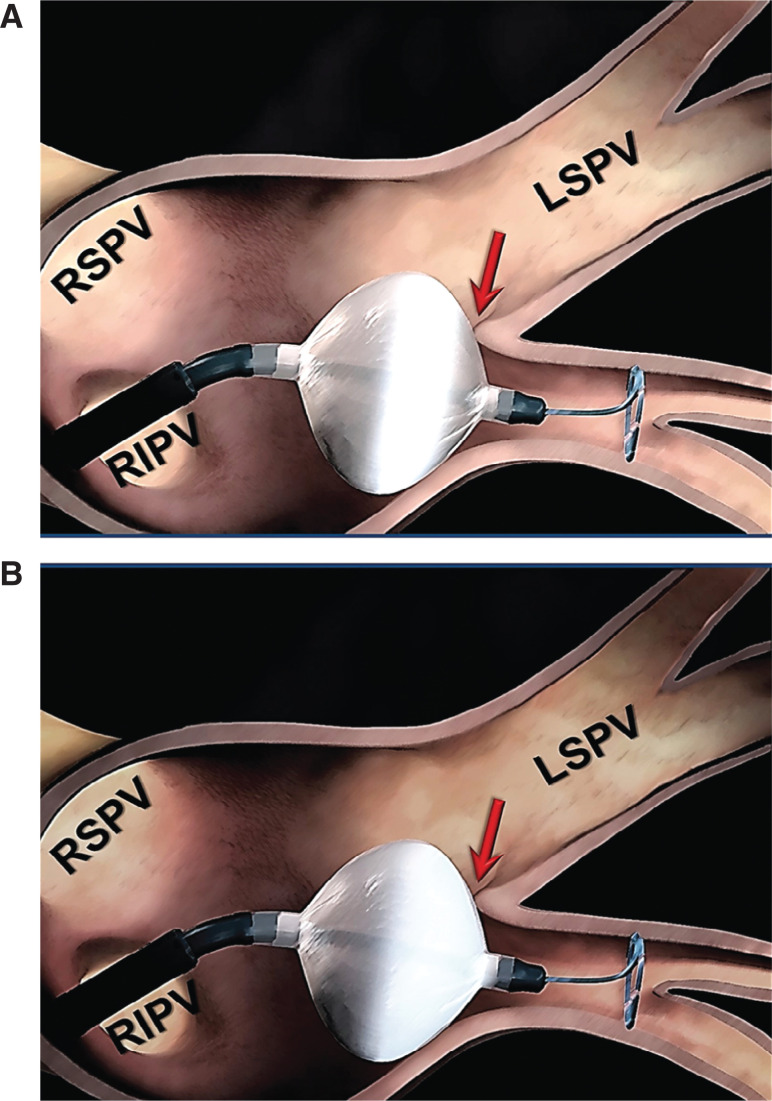
Design differences between the first- and the current-generation cryoballoons. **A:** In the first-generation cryoballoon, the refrigerant distribution comes from four jets placed proximally, yielding a maximal cooling zone that consists of an equatorial band around the balloon’s circumference. Accordingly, optimal balloon positioning does not necessarily equate to optimal tissue contact with the balloon’s maximal cooling zone (arrow). **B:** In the design of the current-generation cryoballoons, the number of injection ports has been increased to eight and they are positioned more distally, resulting in a larger and more uniform zone of freezing. This in turn expands the maximal cooling zone to the entire distal half of the balloon surface. As such, proper balloon alignment can ensure optimal tissue contact with the balloon’s maximal cooling zone (arrow). LSPV: left superior pulmonary vein; RIPV: right inferior pulmonary vein; RSPV: right superior pulmonary vein.

While it is clear that the differences between the study by Reddy et al.^60^ and the more recent experiences, in part, stem from the use of the smaller 23- versus the 28-mm cryoballoon, there is little doubt that the observed differences are also attributed to the design improvements of the current-generation cryoballoons. The incorporation of additional injection ports in the design of the second-/later-generation cryoballoons **(Figure 5)** has led to more optimal and homogenous delivery of cryothermal energy, in turn yielding significantly larger areas of tissue ablation. Not surprisingly, this has been associated with improvements in procedural and clinical efficacy.^63,64^ However, it remains unclear whether the observed clinical improvements are driven by the enhanced ability to create continuous and durable PVI or related to the larger and wider antral areas of circumferential ablation, or perhaps even both.

A study^45^ comparing lesion characteristics and clinical outcomes associated with catheter ablation of AF using the hot balloon (SATAKE HotBalloon; Toray Industries, Tokyo, Japan) versus the current-generation cryoballoon (Arctic Front Advance; Medtronic, Inc) in 71 consecutive patients found that lesions created using the cryoballoon were significantly larger (38.2 ± 12.1 cm^2^ vs. 24.3 ± 8.0 cm^2^). Consistent with this observation, fewer instances of focal touch-up radiofrequency ablation were required with cryoballoon (31% vs. 53%). Similarly, Perrotta et al.^46^ investigated the size of LA isolation following AF ablation using the laser balloon (HeartLight™; CardioFocus, Marlborough, MA, USA) versus the cryoballoon (Arctic Front Advance; Medtronic, Inc) and discovered that total (42 ± 15 vs. 57 ± 14 cm^2^; p = 0.002) and antral (54% ± 10% vs. 65% ± 8%; p = 0.001) surface areas of isolation were both greater with the cryoballoon. Having said that, it should be mentioned that the laser balloon used in this study represented an earlier iteration of this technology and the second/third-generation ablation catheters are believed to provide improved tissue contact and compliance. As such, it remains unknown whether this could have negatively impacted the results of this study. Lastly, in a study comparing the outcomes of cryoballoon ablation again point-by-point, force-sensing radiofrequency, Okumura et al.^47^ showed that the low-voltage areas created using cryoballoon were significantly greater in size and the unexcitable tissue along the ablation line was significantly wider than those created with force-sensing radiofrequency ablation (16.7 ± 5.1 mm vs. 5.3 ± 2.3 mm; p < 0.0001).

### The rationale for nonocclusive cryoballoon ablation (NOCA)

**Figure 6:**
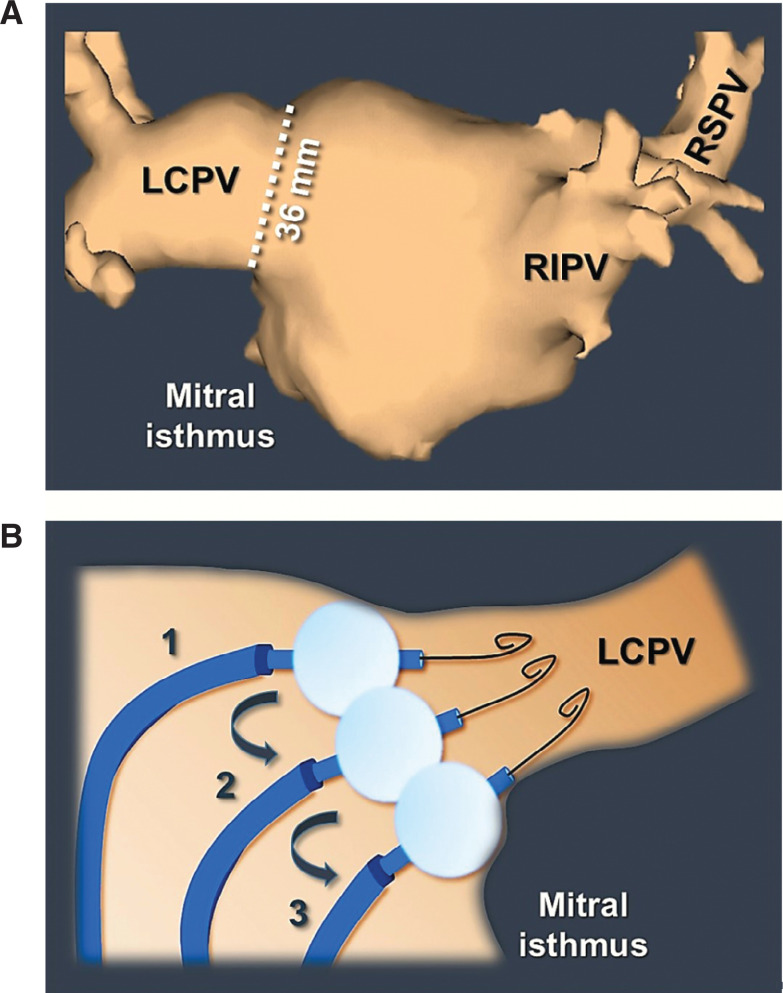
Non-PV occlusive cryoballoon maneuvers for isolation of large-sized PVs. **A:** A cardiac computed tomography image demonstrating a left common PV with an ostium measuring 36 mm in diameter. **B:** When approaching such a vein using a 28-mm cryoballoon, the operator should target the PV with a series of nonocclusive applications to avoid a distal-level (ostial) PVI. In this manner, the PV is ablated antrally in a segmental fashion, as shown in steps 1 to 3. A similar strategy can also be adopted in general when treating large-sized PVs, even in the absence of a “true” common PV ostium. LCPV: large-ostia left common pulmonary vein; RIPV: right inferior pulmonary vein; RSPV: right superior pulmonary vein.

Historically, cryoballoon ablation has been guided by PV occlusion.^65^ Cryothermal effects on cardiac tissue range from reversible ion channel block (ie, electrical dormancy) to permanent cellular injury and death (ie, cellular nonviability). Although cardiac cells can be rendered electrically dormant at temperatures of +20°C to +25°C, it is generally accepted that temperatures between −20°C and −50°C create lethal effects on cardiac cells.^66,67^ Takami et al.^68^ previously demonstrated that the magnitude of PV occlusion using the cryoballoon was an important determinant of durable PVI in a canine model. Similarly, in a clinical study^69^ investigating the procedural and biophysical predictors associated with durable PVI using the second-generation cryoballoon, PV occlusion was found to highly correlate with the significant markers of PVI durability (ie, time to PVI and thaw time). Nevertheless, PV occlusion in itself is likely not a requirement for creating optimal cryolesions. For instance, this is observed when targeting large-sized PVs **(Figure 6)**, as in the case of common PV ostia using a segmental NOCA approach.^70,71^ Although PV occlusion can likely augment the magnitude of the freeze, optimal tissue contact and not necessarily PV occlusion which indirectly implies the same, is believed to be the essential requirement for creating durable cryolesions. This notion is further supported by finite element modeling data.^72^

Meanwhile, the author believes that there is an inherent drawback associated with PV occlusion using the currently available cryoballoons when targeting PVs in patients with persAF. Those with persAF frequently exhibit an enlarged LA and dilated PV antra.^73,74^ Unlike point-by-point radiofrequency, a cryoablation approach guided by PV occlusion using a fixed-diameter cryoballoon (ie, 28 mm) does not permit the operator to adjust the approach accordingly to allow optimal, antral cryoballoon positioning and ablation of the larger PV antra encountered in these patients. Consequently, this approach is more likely to yield an ostial-level PVI in patients with persAF. The author believes that this in part accounts for the reason for diminished success associated with PV occlusion-guided cryoballoon ablation in patients with persAF.^3^ Consistent with this notion, Güler et al.^75^ previously identified PV diameter along with other markers such as persAF and an enlarged LA as significant predictors of AF recurrence following cryoballoon PVI. Along the same lines, Li et al.^76^ identified PV diameter as the strongest independent predictor of long-term AF recurrence following cryoballoon ablation. Furthermore, PV diameter emerged as a more powerful predictor than LA diameter, itself.

### The safety of nonocclusive cryoballoon ablation (NOCA)

When considering a novel ablation technique, it is important to carefully consider the safety of the approach. Due to the close proximity of LAPW to the esophagus, one has to consider the possibility of increased risk of esophageal injury in conjunction with cryo applications required to achieve PWI. However, to date, no increased risk of atrioesophageal fistula has been detected with cryoballoon PWI. Furthermore, a small study recently evaluating the outcomes of cryoballoon PVI + PWI found that interruption of cryoapplications at a luminal esophageal temperature less than 15°C was associated with the absence of esophageal thermal lesions.^77^ As for other potential complications, the only other risk specifically associated with cryoballoon ablation has to do with phrenic nerve injury. It should be emphasized that NOCA or antral cryoballoon applications tend to actually mitigate this risk by avoiding distal placement of the cryoballoon into the PV ostia.^44^ Indeed, this is one of the main reasons behind a higher rate of phrenic nerve injury using balloon-based versus point-by-point radiofrequency ablation strategies.

## The clinical experience with cryoballoon pulmonary vein and posterior wall isolation

**Figure 7:**
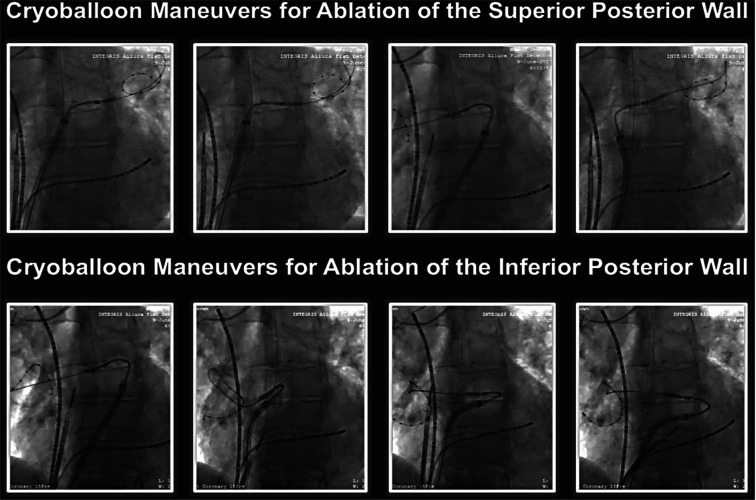
Cryoballoon ablation maneuvers to achieve PWI. Cine images illustrating the cryoballoon positions for ablation of superior **(top)** and inferior **(lower)** segments of the LAPW. To achieve these positions, the guidewire/inner lumen circular catheter is anchored in one of the PVs for stability to create a rail for support of the balloon outside of the PVs. By advancing or retracting the guidewire/inner lumen catheter proximally or distally inside the PV, the operator can maneuver the cryoballoon position along the PW. The superior PVs are typically engaged for ablation of the superior segments of the LAPW and the inferior PVs for ablation of the inferior PW segments.

**Figure 8:**
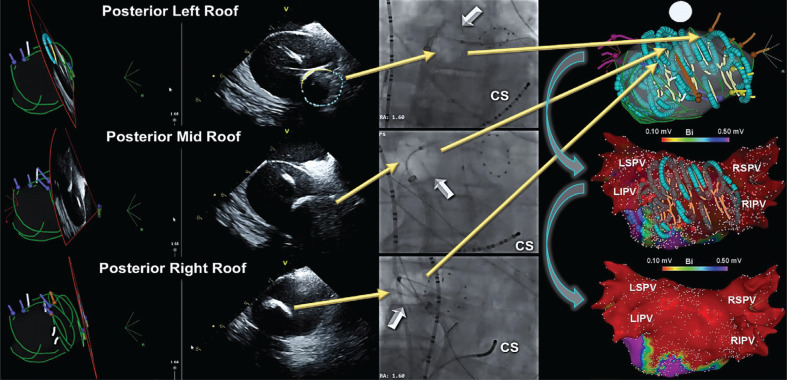
PWI using the cryoballoon guided by ICE and 3D image integration. ICE **(left)** and cine images **(middle)** illustrating cryoballoon positions (arrows) to guide PWI. To visualize the cryoballoon on the LAPW inside the electroanatomic map, ICE 3D image integration can be used. In doing so, the distal surface (maximal cooling zone) of the cryoballoon is traced (turquoise color), whereas the proximal shell as noted by the surface connected to the shaft is depicted in yellow. Consequently, the position of the cryoapplication is directly visualized on the LAPW and recorded within the 3D map **(right)**. As seen, the cryoballoon positions in general correlate well with areas of low voltage recorded on postablation 3D voltage maps. CS: coronary sinus; ICE: intracardiac echocardiography; LIPV: left inferior PV; LSPV: left superior PV; RIPV: right inferior PV; RSPV: right superior PV.

**Figure 9:**
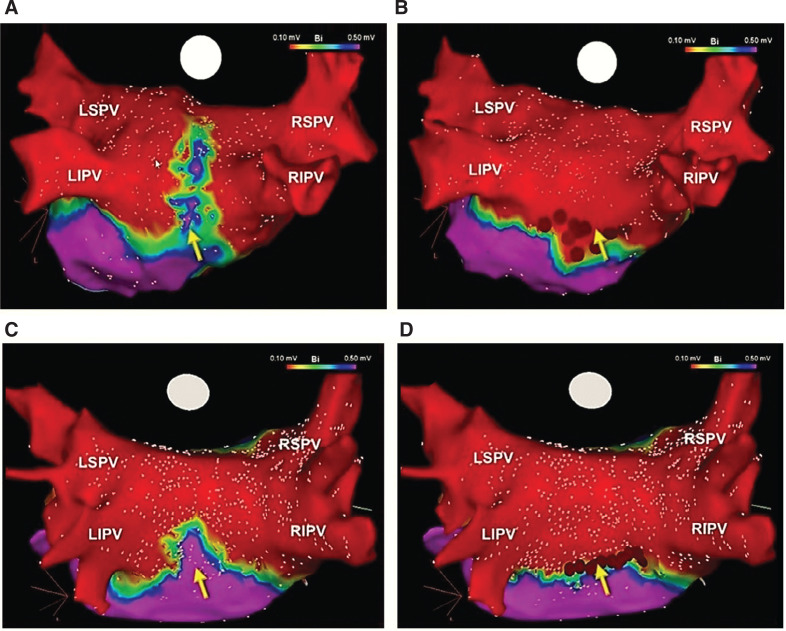
Incomplete PWI using cryoballoon and adjunct radiofrequency ablation. In at least one-third of patients, complete PV component isolation/ablation may not be possible using a 28-mm cryoballoon, specifically in those with a large-sized LA **(A, C)**. The most common site where a gap may be encountered is inferiorly, along the mid portion of the LAPW (arrows). Therefore, as seen in **B** and **D**, adjunct radiofrequency applications (arrows) may be required to successfully complete PWI. LIPV: left inferior PV; LSPV: left superior PV; RIPV: right inferior PV; RSPV: right superior PV.

**Figure 10:**
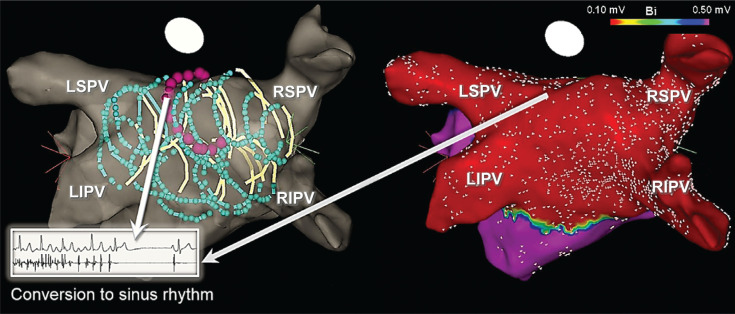
AF termination during cryoballoon PVI + PWI in patients with persAF. Although AF termination and/or conversion to atrial flutters during cryoballoon PVI + PWI is encountered in only 10% to 20% of patients with persAF, when this does occur, it is most commonly observed on the LA roof, as denoted by the magenta-colored application on the ICE-integrated 3D map. ICE: intracardiac echocardiography; LIPV: left inferior PV; LSPV: left superior PV; RIPV: right inferior PV; RSPV: right superior PV.

Using a series of overlapping nonocclusive applications, the cryoballoon offers a simple and facile approach for directly ablating and debulking the LAPW to achieve PVI + PWI **(Figure 7)**. Moreover, this approach can be greatly facilitated through the use of intracardiac echocardiography and 3D image integration **(Figure 8)**. A multicenter, retrospective study previously analyzed the outcomes of PVI + PWI using the second-generation cryoballoon in 222 consecutive patients with symptomatic persAF versus PVI alone.^12^ This study found that acute PWI using this method was feasible in more than two-thirds of the patients without sequelae, whereas adjunct radiofrequency ablation was required to complete this endpoint in the remaining patients **(Figure 9)**. Moreover, as compared with PVI alone, PVI + PWI yielded significantly greater LAPW (77.2% vs. 40.6%; p < 0.001) and total LA (53.3% vs. 36.3%; p < 0.001) isolation, as well as a higher incidence of AF termination and/or conversion to atrial flutters **(Figure 10)**. Adverse events were similar in both groups, whereas recurrence of AF and all atrial arrhythmias was significantly reduced with PVI + PWI at 12 months of follow-up (AF recurrence reduced by ~20%). Moreover, in a Cox regression analysis, PVI + PWI emerged as a significant predictor of freedom from recurrent atrial arrhythmias (hazard ratio: 2.04, 95% confidence interval: 1.15–3.61; p = 0.015). Similar results have been reported in subsequent, retrospective, single-center analyses. For instance, Nishimura et al.^13^ studied the safety and feasibility of cryoballoon PVI + PWI as compared to PVI alone in 100 consecutive patients and found that the former was associated with significantly higher rates of sinus rhythm maintenance at 1 year (80.0% vs. 55.1%; p = 0.01). Nordsieck et al.^14^ also investigated the outcomes of cryoballoon PVI + PWI versus conventional radiofrequency ablation as well as a hybrid surgical approach. These authors found that cryoballoon PVI + PWI was associated with decreased AF recurrence and need for repeat procedures as compared to conventional radiofrequency ablation. Furthermore, cryoballoon PVI + PWI was found to be superior to a hybrid surgical approach with regard to procedural safety and hospital length of stay. Moreover, Iacopino et al.^15^ investigated the value of cryoballoon PVI + PWI in a cohort of patients who underwent a redo procedure for recurrent persAF. They found that this approach was safe and feasible and associated with an 85% freedom from recurrent arrhythmias at 1 year.

**Figure 11:**
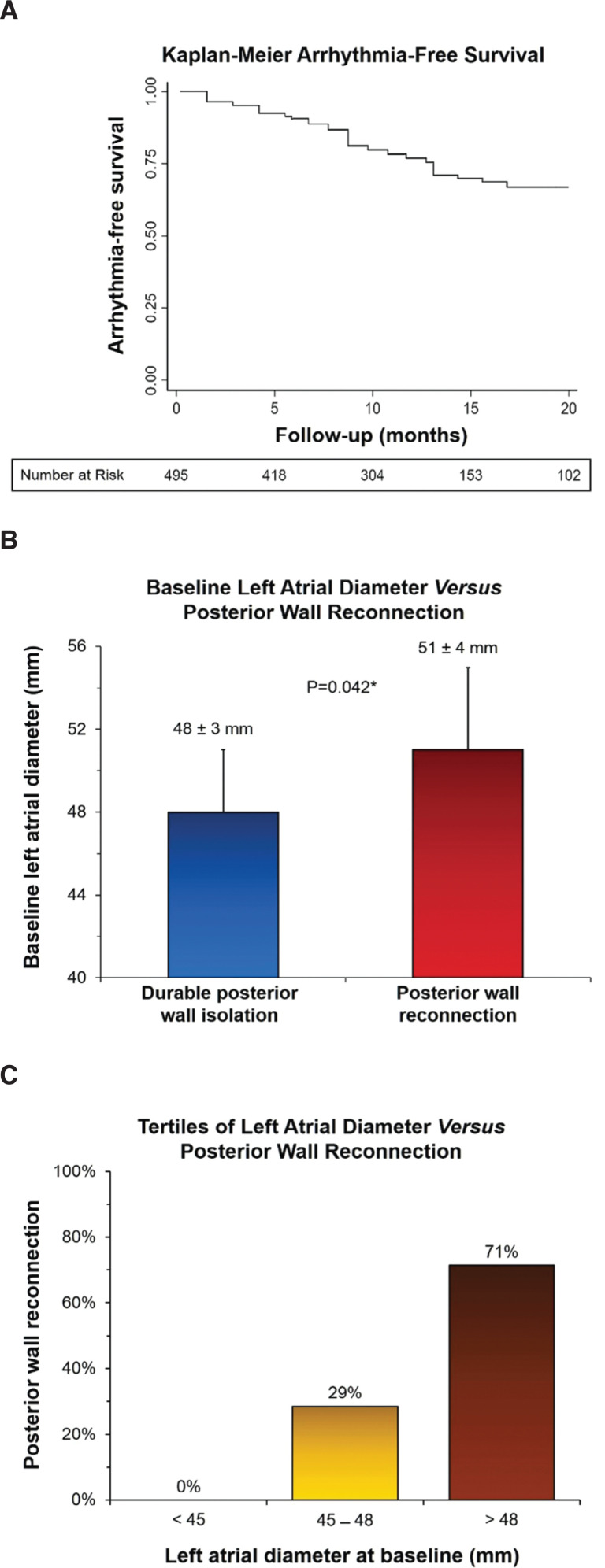
Analysis of LA diameter in patients with versus without LAPW reconnection following cryoballoon PVI + PWI. **A:** A Kaplan–Meier curve illustrating cumulative freedom from recurrent atrial arrhythmias during long-term follow-up in 519 consecutive patients with persAF, who underwent PVI + PWI using the cryoballoon, including the number of patients at risk. **B** In patients who underwent a repeat procedure, the LA diameter was greater in those with versus without LAPW reconnection. **C:** Tertiles of LA diameter (measured in parasternal long-axis view) in patients with LAPW reconnection are shown. As seen, none of the patients with LAPW reconnection exhibited an LA diameter less than 45 mm, whereas 29% demonstrated an LA diameter of 45 to 48 mm and 71% an LA diameter greater than 48 mm. *Significant p-value.

**Figure 12:**
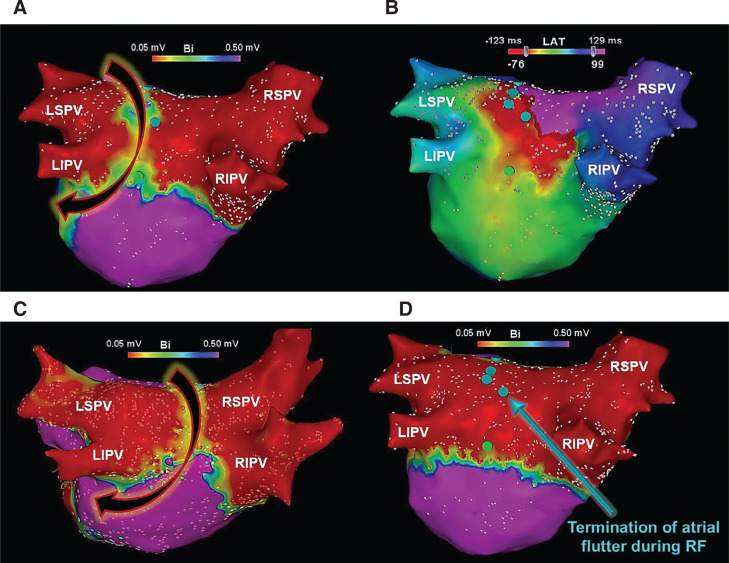
Electroanatomic maps created at repeat procedures. 3D voltage **(A)** and activation **(B)** maps demonstrating partial LAPW reconnection and an atypical atrial flutter using the LAPW/roof for reentry, following a prior cryoballoon PVI + PWI. Radiofrequency applications along the LAPW (turquoise lesions) resulted in termination of the tachycardia, rendering it noninducible. **C:** Another 3D voltage map in a patient who underwent a previous cryoballoon PVI + PWI, demonstrating partial LAPW conduction recovery in a patient referred for repeat ablation. Although this patient did not present clinically with a related atrial flutter, a reentrant LA roof tachycardia was inducible at electrophysiology study. **D:** Radiofrequency applications applied to the LAPW (turquoise lesions) terminated the tachycardia and rendered it no longer inducible. Both patients have maintained sinus rhythm off antiarrhythmic therapy during long-term follow-up. LIPV: left inferior PV; LSPV: left superior PV; RF: radiofrequency; RIPV: right inferior PV; RSPV: right superior PV.

In a more recent study,^78^ the durability of PVI + PWI using the 28-mm cryoballoon was investigated in 81 consecutive patients referred to undergo repeat ablation for arrhythmia recurrence. The analysis showed durable PWI in 67 of 81 patients (82.7%) during 18 ± 4 months of follow-up. It also found that LA diameter (measured in parasternal long-axis view) represented a significant predictor for the need for adjunct radiofrequency ablation, particularly an LA diameter greater than 48 mm. Additionally, those with LAPW reconnection exhibited larger LA diameters, such that, none of the patients with PW reconnection exhibited an LA diameter measuring less than 45 mm, whereas 29% demonstrated an LA diameter of 45 to 48 mm and 71% an LA diameter greater than 48 mm (negative predictive value: 89.7%) **(Figure 11)**. An atypical LAPW/roof flutter represented the third most common cause of arrhythmia recurrence and virtually every patient with LAPW reconnection exhibited such an arrhythmia **(Figure 12)**. In a more recent multicenter, prospective, randomized-controlled trial (ClinicalTrials.gov #NCT03057548), once again a 20% reduction in AF recurrence was observed at 12 months of follow-up with PVI + PWI as compared to PVI alone (25.5% vs. 45.5%; p = 0.028), in patients with symptomatic persAF.^44^ Adverse events were similar, but approximately 45% required adjunct radiofrequency ablation to complete PWI. PVI + PWI was also associated with fewer intraprocedural cardioversions. Furthermore, PVI + PWI yielded a much greater reduction in AF recurrence in patients with versus without LAPW low-voltage areas recorded at baseline (11.8% vs. 35.6%; p < 0.05). Lastly, PVI + PWI emerged as a significant predictor of freedom from recurrent AF (odds ratio: 3.67, 95% confidence interval: 1.44–9.34; p = 0.006). The validity of these studies and their findings will be ultimately tested on a larger scale in the PIVoTAL-IDE (LAPW and PV Isolation Using Cryoballoon for Treatment of persAF) trial (ClinicalTrials.gov #NCT04505163). This study,^79^ which is currently underway, is a large, prospective, multicenter, randomized-controlled IDE trial (G190171) with the aim to precisely evaluate the acute and long-term outcomes of PVI + PWI versus PVI alone using the cryoballoon in patients with symptomatic persAF. Undoubtedly, future modifications and evolutions in catheter design, including larger balloons and non-fixed/variable diameters, will help to significantly facilitate and enhance the NOCA approach to catheter ablation and isolation of PVs and extra-PV structures.

## References

[1] Haïssaguerre M, Jaïs P, Shah DC (1998). Spontaneous initiation of atrial fibrillation by ectopic beats originating in the pulmonary veins. N Engl J Med.

[2] Brooks AG, Stiles MK, Laborderie J (2010). Outcomes of long-standing persistent atrial fibrillation ablation: a systematic review. Heart Rhythm.

[3] Su WW, Reddy VY, Bhasin K (2020). Cryoballoon ablation of pulmonary veins for persistent atrial fibrillation: results from the multicenter STOP Persistent AF trial. Heart Rhythm.

[4] Lappe JM, Cutler MJ, Day JD, Bunch TJ (2016). Ablation for persistent atrial fibrillation—is there a role for more than PVI?. Curr Treat Options Cardiovasc Med.

[5] Elbatran AI, Anderson RH, Mori S, Saba MM (2019). The rationale for isolation of the left atrial pulmonary venous component to control atrial fibrillation: a review article. Heart Rhythm.

[6] Badhwar N, Tschopp DR, Lee RJ (2015). Surgical and concomitant epicardial-endocardial (hybrid) ablation of persistent and long-standing persistent atrial fibrillation. Curr Probl Cardiol.

[7] Cox JL, Canavan TE, Schuessler RB (1991). The surgical treatment of atrial fibrillation. II. Intraoperative electrophysiologic mapping and description of the electrophysiologic basis of atrial flutter and atrial fibrillation. J Thorac Cardiovasc Surg.

[8] Cox JL, Ad N, Palazzo T (2000). Current status of the maze procedure for the treatment of atrial fibrillation. Semin Thorac Cardiovasc Surg.

[9] Segerson NM, Daccarett M, Badger TJ (2010). Magnetic resonance imaging-confirmed ablative debulking of the left atrial posterior wall and septum for treatment of persistent atrial fibrillation: rationale and initial experience. J Cardiovasc Electrophysiol.

[10] He X, Zhou Y, Chen Y, Wu L, Huang Y, He J (2016). Left atrial posterior wall isolation reduces the recurrence of atrial fibrillation: a meta-analysis. J Interv Card Electrophysiol..

[11] Bai R, Di Biase L, Mohanty P (2016). Proven isolation of the pulmonary vein antrum with or without left atrial posterior wall isolation in patients with persistent atrial fibrillation. Heart Rhythm.

[12] Aryana A, Baker JH, Espinosa Ginic MA (2018). Posterior wall isolation using the cryoballoon in conjunction with pulmonary vein ablation is superior to pulmonary vein isolation alone in patients with persistent atrial fibrillation: a multicenter experience. Heart Rhythm.

[13] Nishimura T, Yamauchi Y, Aoyagi H (2019). The clinical impact of the left atrial posterior wall lesion formation by the cryoballoon application for persistent atrial fibrillation: feasibility and clinical implications. J Cardiovasc Electrophysiol.

[14] Nordsieck E, Zhang XJ, Malhotra P, Fan D, Pezeshkian NG, Srivatsa UN (2019). Comparison of cryoballoon and hybrid surgical posterior wall isolation for persistent atrial fibrillation to conventional ablation. J Atr Fibrillation.

[15] Iacopino S, Paparella G, Capulzini L (2019). Posterior box isolation as an adjunctive ablation strategy during repeat ablation with the second-generation cryoballoon for recurrence of persistent atrial fibrillation: 1-year follow-up. J Interv Card Electrophysiol.

[16] Proietti R, Santangeli P, Di Biase L (2014). Comparative effectiveness of wide antral versus ostial pulmonary vein isolation: a systematic review and meta-analysis. Circ Arrhythm Electrophysiol.

[17] Nathan H, Eliakim M (1966). The junction between the left atrium and the pulmonary veins. An anatomic study of human hearts. Circulation.

[18] Bai R (2016). Left atrial posterior wall isolation: the icing on the cake. J Interv Card Electrophysiol.

[19] Anderson RH, Brown NA, Moorman AFM (2006). Development and structures of the venous pole of the heart. Dev Dyn.

[20] Webb S, Kanani M, Anderson RH, Richardson MK, Brown NA (2001). Development of the human pulmonary vein and its incorporation in the morphologically left atrium. Cardiol Young.

[21] Jones SA, Yamamoto M, Tellez JO (2008). Distinguishing properties of cells from the myocardial sleeves of the pulmonary veins: a comparison of normal and abnormal pacemakers. Circ Arrhythm Electrophysiol.

[22] Roberts-Thomson KC, Stevenson I, Kistler PM (2009). The role of chronic atrial stretch and atrial fibrillation on posterior left atrial wall conduction. Heart Rhythm.

[23] Corradi D, Callegari S, Maestri R (2012). Differential structural remodeling of the left-atrial posterior wall in patients affected by mitral regurgitation with or without persistent atrial fibrillation: a morphological and molecular study. J Cardiovasc Electrophysiol.

[24] Markides V, Schilling RJ, Ho SY, Chow AW, Davies DW, Peters NS (2003). Characterization of left atrial activation in the intact human heart. Circulation.

[25] Suenari K, Chen YC, Kao YH (2011). Discrepant electrophysiological characteristics and calcium homeostasis of left atrial anterior and posterior myocytes. Basic Res Cardiol.

[26] Ehrlich JR, Cha T, Zhang L (2003). Cellular electrophysiology of canine pulmonary vein cardiomyocytes: action potential and ionic current properties. J Physiol.

[27] Verheule S, Tuyls E, Van Hunnik A (2010). Fibrillatory conduction in the atrial free walls of goats in persistent and permanent atrial fibrillation. Circ Arrhythmia Electrophysiol.

[28] Tahir KS, Mounsey JP, Hummel JP (2018). Posterior wall isolation in atrial fibrillation ablation. J Innov Card Rhythm Manag.

[29] Allessie MA, De Groot NMS, Houben RPM (2010). Electropathological substrate of long-standing persistent atrial fibrillation in patients with structural heart disease: longitudinal dissociation. Circ Arrhythm Electrophysiol.

[30] Mandapati R, Skanes A, Chen J, Berenfeld O, Jalife J (2000). Stable microreentrant sources as a mechanism of atrial fibrillation in the isolated sheep heart. Circulation.

[31] Spector P (2013). Principles of cardiac electric propagation and their implications for re-entrant arrhythmias. Circ Arrhythm Electrophysiol.

[32] Narayan SM, Krummen DE, Clopton P, Shivkumar K, Miller JM (2013). Direct or coincidental elimination of stable rotors or focal sources may explain successful atrial fibrillation ablation: on-treatment analysis of the CONFIRM trial (Conventional ablation for AF with or without focal impulse and rotor modulation). J Am Coll Cardiol.

[33] Nademanee K, McKenzie J, Kosar E (2004). A new approach for catheter ablation of atrial fibrillation: mapping of the electrophysiologic substrate. J Am Coll Cardiol.

[34] Lin WS, Tai CT, Hsieh MH (2003). Catheter ablation of paroxysmal atrial fibrillation initiated by non-pulmonary vein ectopy. Circulation.

[35] Kalifa J, Maixent JM, Chalvidan T (2008). Energetic metabolism during acute stretch-related atrial fibrillation. Mol Cell Biochem.

[36] Hunter RJ, Liu Y, Lu Y, Wang W, Schilling RJ (2012). Left atrial wall stress distribution and its relationship to electrophysiologic remodeling in persistent atrial fibrillation. Circ Arrhythm Electrophysiol.

[37] Pison L, La Meir M, van Opstal J, Blaauw Y, Maessen J, Crijns HJ (2012). Hybrid thoracoscopic surgical and transvenous catheter ablation of atrial fibrillation. J Am Coll Cardiol.

[38] Lim TW, Koay CH, See VA (2012). Single-ring posterior left atrial (box) isolation results in a different mode of recurrence compared with wide antral pulmonary vein isolation on long-term follow-up: longer atrial fibrillation-free survival time but similar survival time free of any atrial arrhythmia. Circ Arrhythm Electrophysiol.

[39] Reddy VY, Neuzil P, D’Avila A, Ruskin JN (2008). Isolating the posterior left atrium and pulmonary veins with a “box” lesion set: use of epicardial ablation to complete electrical isolation. J Cardiovasc Electrophysiol.

[40] Higuchi S, Sohara H, Nakamura Y (2016). Is it necessary to achieve a complete box isolation in the case of frequent esophageal temperature rises? Feasibility of shifting to a partial box isolation strategy for patients with non-paroxysmal atrial fibrillation. J Cardiovasc Electrophysiol.

[41] Kim J, Shin SY, Na JO (2015). Does isolation of the left atrial posterior wall improve clinical outcomes after radiofrequency catheter ablation for persistent atrial fibrillation? A prospective randomized clinical trial. Int J Cardiol.

[42] Sou T, Yukiko N, Tetsuji S, Kazuaki C, Katsuhiko I, Taijiro S (2004). Atrial contraction after surgical isolation of the left atrial posterior wall concomitant with mitral valve replacement. Circ J.

[43] Zhang X, Beri N, Malhotra P (2020). Posterior wall isolation for atrial fibrillation: effects on echocardiographic parameters of cardiac function. J Atr Fibrillation.

[44] Aryana A, Allen SL, Pujara DK (2021). Concomitant pulmonary vein and posterior wall isolation using cryoballoon with adjunct radiofrequency in persistent atrial fibrillation. JACC Clin Electrophysiol.

[45] Nagashima K, Okumura Y, Watanabe I (2018). Hot balloon versus cryoballoon ablation for atrial fibrillation: lesion characteristics and efficacy. Circ Arrhythm Electrophysiol.

[46] Perrotta L, Konstantinou A, Bordignon S (2017). What is the acute antral lesion size after pulmonary vein isolation using different balloon ablation technologies?. Circ J.

[47] Okumura Y, Watanabe I, Iso K (2017). Mechanistic insights into durable pulmonary vein isolation achieved by second-generation cryoballoon ablation. J Atr Fibrillation.

[48] Reddy VY, Sediva L, Petru J (2015). Durability of pulmonary vein isolation with cryoballoon ablation: results from the Sustained PV Isolation with Arctic Front Advance (SUPIR) study. J Cardiovasc Electrophysiol.

[49] Piccini JP, Braegelmann KM, Simma S, Koneru JN, Ellenbogen KA (2020). Risk of atrioesophageal fistula with cryoballoon ablation of atrial fibrillation. Heart Rhythm O^2^.

[50] Ripley KL, Gage AA, Olsen DB, Van Vleet JF, Lau C-P, Tse H-F (2007). Time course of esophageal lesions after catheter ablation with cryothermal and radiofrequency ablation: implication for atrio-esophageal fistula formation after catheter ablation for atrial fibrillation. J Cardiovasc Electrophysiol.

[51] Squara F, Zhao A, Marijon E (2015). Comparison between radiofrequency with contact force-sensing and second-generation cryoballoon for paroxysmal atrial fibrillation catheter ablation: a multicentre European evaluation. Europace.

[52] Schmidt M, Nölker G, Marschang H (2008). Incidence of oesophageal wall injury post-pulmonary vein antrum isolation for treatment of patients with atrial fibrillation. Europace.

[53] Di Biase L, Saenz LC, Burkhardt DJ (2009). Esophageal capsule endoscopy after radiofrequency catheter ablation for atrial fibrillation: documented higher risk of luminal esophageal damage with general anesthesia as compared with conscious sedation. Circ Arrhythm Electrophysiol.

[54] Tilz RR, Chun KRJ, Metzner A (2010). Unexpected high incidence of esophageal injury following pulmonary vein isolation using robotic navigation. J Cardiovasc Electrophysiol.

[55] Metzner A, Burchard A, Wohlmuth P (2013). Increased incidence of esophageal thermal lesions using the second-generation 28-mm cryoballoon. Circ Arrhythm Electrophysiol.

[56] Fürnkranz A, Chun KRJ, Metzner A (2010). Esophageal endoscopy results after pulmonary vein isolation using the single big cryoballoon technique. J Cardiovasc Electrophysiol.

[57] Fürnkranz A, Bordignon S, Böhmig M (2015). Reduced incidence of esophageal lesions by luminal esophageal temperature-guided second-generation cryoballoon ablation. Heart Rhythm.

[58] Sarairah SY, Woodbury B, Methachittiphan N, Tregoning DM, Sridhar AR, Akoum N (2020). Esophageal thermal injury following cryoballoon ablation for atrial fibrillation. JACC Clin Electrophysiol.

[59] Cai D, Liu Q, Shehata M (2020). Esophageal contraction during cryoablation: a possible protective mechanism. Pacing Clin Electrophysiol.

[60] Reddy VY, Neuzil P, d’Avila A (2008). Balloon catheter ablation to treat paroxysmal atrial fibrillation: what is the level of pulmonary venous isolation?. Heart Rhythm.

[61] Chierchia GB, de Asmundis C, Sorgente A (2011). Anatomical extent of pulmonary vein isolation after cryoballoon ablation for atrial fibrillation: comparison between the 23 and 28 mm balloons. J Cardiovasc Med (Hagerstown).

[62] Kenigsberg DN, Martin N, Lim HW, Kowalski M, Ellenbogen KA (2015). Quantification of the cryoablation zone demarcated by pre- and postprocedural electroanatomic mapping in patients with atrial fibrillation using the 28-mm second-generation cryoballoon. Heart Rhythm.

[63] Fürnkranz A, Bordignon S, Dugo D (2014). Improved 1-year clinical success rate of pulmonary vein isolation with the second-generation cryoballoon in patients with paroxysmal atrial fibrillation. J Cardiovasc Electrophysiol.

[64] Aryana A, Morkoch S, Bailey S (2014). Acute procedural and cryoballoon characteristics from cryoablation of atrial fibrillation using the first- and second-generation cryoballoon: a retrospective comparative study with follow-up outcomes. J Interv Card Electrophysiol.

[65] Aryana A, Kenigsberg DN, Kowalski M (2017). Cryo-DOSING Investigators. Verification of a novel atrial fibrillation cryoablation dosing algorithm guided by time-to-pulmonary vein isolation: results from the Cryo-DOSING Study (Cryoballoon-ablation DOSING Based on the Assessment of Time-to-Effect and Pulmonary Vein Isolation Guidance). Heart Rhythm.

[66] Aufderheide T (2007). Etiology, Electrophysiology, and Myocardial Mechanics of Pulseless Electrical Activity. Cardiac Arrest, the Science and Practice of Resuscitation Medicine.

[67] Gage AA, Baust JM, Baust JG (2009). Experimental cryosurgery investigations in vivo. Cryobiology.

[68] Takami M, Misiri J, Lehmann HI (2015). Spatial and time-course thermodynamics during pulmonary vein isolation using the second-generation cryoballoon in a canine in vivo model. Circ Arrhythm Electrophysiol.

[69] Aryana A, Mugnai G, Singh SM (2016). Procedural and biophysical indicators of durable pulmonary vein isolation during cryoballoon ablation of atrial fibrillation. Heart Rhythm.

[70] Heeger CH, Tscholl V, Wissner E (2017). Acute efficacy, safety, and long-term clinical outcomes using the second-generation cryoballoon for pulmonary vein isolation in patients with a left common pulmonary vein: a multicenter study. Heart Rhythm.

[71] Ströker E, Takarada K, de Asmundis C (2017). Second-generation cryoballoon ablation in the setting of left common pulmonary veins: procedural findings and clinical outcome. Heart Rhythm.

[72] Handler M, Fischer G, Seger M, Kienast R, Hanser F, Baumgartner C (2015). Simulation and evaluation of freeze-thaw cryoablation scenarios for the treatment of cardiac arrhythmias. Biomed Eng Online.

[73] Mlcochová H, Tintera J, Porod V, Peichl P, Cihák R, Kautzner J (2005). Magnetic resonance angiography of pulmonary veins: implications for catheter ablation of atrial fibrillation. Pacing Clin Electrophysiol.

[74] Wozakowska-Kaplon B (2005). Changes in left atrial size in patients with persistent atrial fibrillation: a prospective echocardiographic study with a 5-year follow-up period. Int J Cardiol.

[75] Güler E, Güler GB, Demir GG (2015). Effect of pulmonary vein anatomy and pulmonary vein diameters on outcome of cryoballoon catheter ablation for atrial fibrillation. Pacing Clin Electrophysiol.

[76] Li B, Ma H, Guo H (2019). Pulmonary vein parameters are similar or better predictors than left atrial diameter for paroxysmal atrial fibrillation after cryoablation. Braz J Med Biol Res.

[77] Osório TG, Iacopino S, Coutiño HE (2019). Evaluation of the luminal esophageal temperature behavior during left atrium posterior wall ablation by means of second-generation cryoballoon. J Interv Card Electrophysiol.

[78] Aryana A, Pujara DK, Baker JH (2020). Long-term durability of posterior wall isolation using the cryoballoon in patients with persistent atrial fibrillation: a multicenter analysis of repeat catheter ablations. J Interv Card Electrophysiol.

[79] Aryana A, Pujara DK, Allen SL (2020). Left atrial posterior wall isolation in conjunction with pulmonary vein isolation using cryoballoon for treatment of persistent atrial fibrillation (PIVoTAL): study rationale and design. J Interv Card Electrophysiol.

